# *trans*-4-(Tosyl­oxymeth­yl)cyclo­hexane­carboxylic acid

**DOI:** 10.1107/S1600536807068572

**Published:** 2008-01-09

**Authors:** Qing-Rong Qi, Wen-Cai Huang, Hu Zheng

**Affiliations:** a Department of Medicinal Chemistry, West China School of Pharmacy, Sichuan University, Chengdu 610041, People’s Republic of China; b Department of Pharmaceutical and Bioengineering, School of Chemical Engineering, Sichuan University, Chengdu 610065, People’s Republic of China

## Abstract

The title compound, C_15_H_20_O_5_S, is an inter­mediate in the synthesis of a new type of poly(amido­amine) (PAMAM) dendrimer. The cyclo­hexane ring exhibits a chair conformation, with C—C bond lengths in the range 1.518 (3)–1.531 (3) Å and C—C—C angles in the range 110.45 (19)–112.09 (19)°; these agree well with the values in other cyclo­hexane derivatives described in the literature. In the crystal structure, adjacent mol­ecules are linked by O—H⋯·O hydrogen bonds. The H atoms of the methyl group are disordered equally over two positions.

## Related literature

For related literature, see: Ahmed *et al.* (2001 ▶); Bucourt & Hainaut (1965 ▶); Dunitz & Strickler (1966 ▶); Grabchev *et al.* (2003 ▶); Luger *et al.* (1972 ▶); Wang *et al.* (2004 ▶); van Koningsveld & Jansen (1984 ▶).
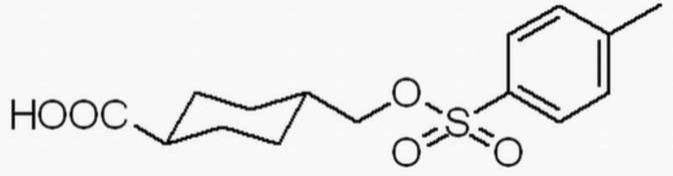


## Experimental

### 

#### Crystal data

C_15_H_20_O_5_S
*M* *_r_* = 312.37
Triclinic, 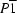

*a* = 5.9006 (5) Å
*b* = 7.0880 (9) Å
*c* = 20.2754 (18) Å
α = 90.371 (3)°
β = 97.479 (2)°
γ = 111.222 (2)°
*V* = 782.44 (14) Å^3^
*Z* = 2
Mo *K*α radiation
μ = 0.23 mm^−1^
*T* = 293 (2) K
0.53 × 0.48 × 0.12 mm

#### Data collection

Rigaku R-AXIS RAPID diffractometer
Absorption correction: multi-scan (*ABSCOR*; Higashi, 1995 ▶) *T*_min_ = 0.890, *T*_max_ = 0.974
7685 measured reflections
3562 independent reflections
2442 reflections with *I* > 2σ(*I*)
*R*_int_ = 0.024

#### Refinement

*R*[*F* ^2^ > 2σ(*F* ^2^)] = 0.044
*wR*(*F* ^2^) = 0.179
*S* = 1.01
3562 reflections
195 parameters
H atoms treated by a mixture of independent and constrained refinement
Δρ_max_ = 0.30 e Å^−3^
Δρ_min_ = −0.48 e Å^−3^

### 

Data collection: *RAPID-AUTO* (Rigaku, 2004 ▶); cell refinement: *RAPID-AUTO*; data reduction: *RAPID-AUTO*; program(s) used to solve structure: *SHELXS97* (Sheldrick, 2008 ▶); program(s) used to refine structure: *SHELXL97* (Sheldrick, 2008 ▶); molecular graphics: *SHELXTL* (Sheldrick, 1997 ▶); software used to prepare material for publication: *SHELXTL*.

## Supplementary Material

Crystal structure: contains datablocks global, I. DOI: 10.1107/S1600536807068572/wn2233sup1.cif

Structure factors: contains datablocks I. DOI: 10.1107/S1600536807068572/wn2233Isup2.hkl

Additional supplementary materials:  crystallographic information; 3D view; checkCIF report

## Figures and Tables

**Table 1:** Hydrogen-bond geometry (Å, °)

*D*—H⋯*A*	*D*—H	H⋯*A*	*D*⋯*A*	*D*—H⋯*A*
O4—H4O⋯O5^i^	0.89 (4)	1.76 (4)	2.654 (3)	178 (3)

Symmetry code: (i) 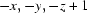.

**Fig. 1.**
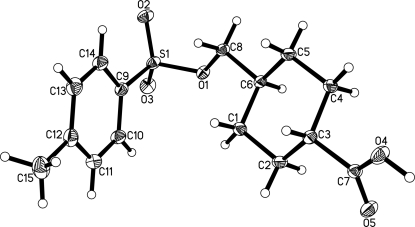
The molecular structure of the title compound, with displacement ellipsoids drawn at the 20% probability level.

**Fig. 2.**
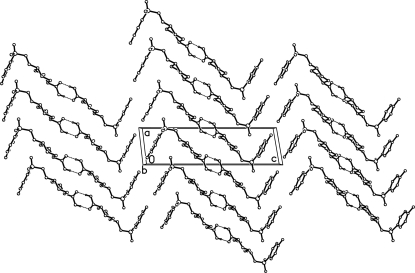
A packing diagram of the title compound. Intermolecular hydrogen bonds are shown as dashed lines.

## supplementary crystallographic information

### Comment

PAMAM (poly(amidoamine)) dendrimers have attracted much interest for their symmetry, high degree of branching and high density of terminal functional groups, which can participate in different reactions. The modification of the periphery of PAMAM dendrimers, aimed to change their physical or chemical properties, have been reported recently (Grabchev *et al.*,2003; Ahmed *et al.*,2001; Wang *et al.*,2004). To improve the lipophilicity of PAMAM dendrimers and provide a new type of linker with special stereostructure, a series of cyclohexane derivatives were synthesized. In our synthetic work on PAMAM dendrimers, we obtained the title compound, and report here its crystal structure.

The crystal structure shows that molecules are linked by O—H···.O hydrogen bonds and the cyclohexane ring exists in the chair conformation. The mean C—C bond length of the cyclohexane ring is 1.524 (3) Å, which is close to the value in *trans*-1,4-cyclohexane dicarboxylic acid (1.523 (3) Å; Luger *et al.*, 1972). The mean endocyclic angle is 111.3 (2)°, which is close to the value for an ideal cyclohexane ring, (C—C—C 111.1°; Bucourt & Hainaut, 1965) and the mean value in *trans*-1,4-cyclohexanedicarboxylic acid (111.4 (4)°; Dunitz & Strickler, 1966; Luger *et al.*, 1972).

### Experimental

*trans*-4-(Methoxycarbonyl)cyclohexanemethanol (10 mmol), triethylamine (10 mmol) and a small amount of trimethylamine hydrochloride were suspended in dichloromethane (20 ml), and *p*-toluenesulfonyl chloride (11 mmol) was added dropwise with vigorous stirring at room temperature; after 1 h the reaction was quenched by addition of water. The organic layer which separated was evaporated to give an oil and the oil was hydrolyzed in a methanol and aqueous NaOH (11 mmol) solution for 5 h at 323 K. The title compound was then obtained by acidification with hydrochloric acid and recrystallized from acetone. Colorless crystals suitable for X-ray analysis were obtained by slow evaporation of a cyclohexane and acetone solution at room temperature.

### Refinement

The carboxyl H was located in a difference Fourier map and refined freely to an O—H value of 0.89 (4) Å. The other H atoms were placed in calculated positions and refined in the riding model approximation, with C—H = 0.93, 0.96, 0.97, or 0.98 Å for benzene, methyl, methylene or methine H atoms, respectively. For carbon-bound H atoms, *U*_iso_(H) = 1.2*U*_eq_(C). The H atoms of the methyl group are disordered equally over two positions.

### Crystal data

**Table:**

C_15_H_20_O_5_S	*Z* = 2
*M**_r_* = 312.37	*F*_000_ = 332
Triclinic, *P*1	*D*_x_ = 1.326 Mg m^−^^3^
*a* = 5.9006 (5) Å	Mo *K*α radiation λ = 0.71073 Å
*b* = 7.0880 (9) Å	Cell parameters from 5250 reflections
*c* = 20.2754 (18) Å	θ = 3.1–27.5º
α = 90.371 (3)º	µ = 0.23 mm^−^^1^
β = 97.479 (2)º	*T* = 293 (2) K
γ = 111.222 (2)º	Block, colourless
*V* = 782.44 (14) Å^3^	0.53 × 0.48 × 0.12 mm

### Data collection

**Table:**

Rigaku R-AXIS RAPID diffractometer	3562 independent reflections
Radiation source: Rotating Anode	2442 reflections with *I* > 2σ(*I*)
Monochromator: graphite	*R*_int_ = 0.024
*T* = 293(2) K	θ_max_ = 27.5º
ω scans	θ_min_ = 3.1º
Absorption correction: multi-scan(ABSCOR; Higashi, 1995)	*h* = −6→7
*T*_min_ = 0.890, *T*_max_ = 0.974	*k* = −9→9
7685 measured reflections	*l* = −26→26

### Refinement

**Table:**

Refinement on *F*^2^	Hydrogen site location: inferred from neighbouring sites
Least-squares matrix: full	H atoms treated by a mixture of independent and constrained refinement
*R*[*F*^2^ > 2σ(*F*^2^)] = 0.044	*w* = 1/[σ^2^(*F*_o_^2^) + (0.1018*P*)^2^ + 0.285*P*] where *P* = (*F*_o_^2^ + 2*F*_c_^2^)/3
*wR*(*F*^2^) = 0.179	(Δ/σ)_max_ < 0.001
*S* = 1.01	Δρ_max_ = 0.30 e Å^−^^3^
3562 reflections	Δρ_min_ = −0.48 e Å^−^^3^
195 parameters	Extinction correction: SHELXL97 (Sheldrick, 1997a), Fc^*^=kFc[1+0.001xFc^2^λ^3^/sin(2θ)]^-1/4^
Primary atom site location: structure-invariant direct methods	Extinction coefficient: 0.047 (7)
Secondary atom site location: difference Fourier map	

### Special details

**Table:**

Geometry. All e.s.d.'s (except the e.s.d. in the dihedral angle between two l.s. planes) are estimated using the full covariance matrix. The cell e.s.d.'s are taken into account individually in the estimation of e.s.d.'s in distances, angles and torsion angles; correlations between e.s.d.'s in cell parameters are only used when they are defined by crystal symmetry. An approximate (isotropic) treatment of cell e.s.d.'s is used for estimating e.s.d.'s involving l.s. planes.
Refinement. Refinement of *F*^2^ against ALL reflections. The weighted *R*-factor *wR* and goodness of fit *S* are based on *F*^2^, conventional *R*-factors *R* are based on *F*, with *F* set to zero for negative *F*^2^. The threshold expression of *F*^2^ > σ(*F*^2^) is used only for calculating *R*-factors(gt) *etc*. and is not relevant to the choice of reflections for refinement. *R*-factors based on *F*^2^ are statistically about twice as large as those based on *F*, and *R*- factors based on ALL data will be even larger.

### Fractional atomic coordinates and isotropic or equivalent isotropic displacement parameters (Å^2^)

**Table:**

	*x*	*y*	*z*	*U*_iso_*/*U*_eq_	Occ. (<1)
S1	0.95047 (11)	0.96589 (9)	0.20554 (3)	0.0486 (2)	
O1	0.8978 (3)	0.9010 (3)	0.27746 (8)	0.0470 (4)	
O2	0.8958 (4)	1.1432 (3)	0.19236 (10)	0.0610 (5)	
O3	1.1925 (3)	0.9708 (3)	0.20334 (10)	0.0655 (5)	
O4	−0.1018 (4)	0.1728 (3)	0.44904 (11)	0.0642 (6)	
O5	0.2394 (4)	0.1042 (3)	0.46193 (10)	0.0624 (5)	
C1	0.5304 (5)	0.5148 (3)	0.31242 (13)	0.0497 (6)	
H1A	0.4091	0.4897	0.2729	0.060*	
H1B	0.6801	0.5106	0.2988	0.060*	
C2	0.4366 (5)	0.3497 (4)	0.36070 (13)	0.0518 (6)	
H2A	0.3981	0.2181	0.3383	0.062*	
H2B	0.5646	0.3666	0.3980	0.062*	
C3	0.2093 (4)	0.3565 (3)	0.38646 (11)	0.0431 (5)	
H3	0.0790	0.3290	0.3484	0.052*	
C4	0.2559 (5)	0.5666 (3)	0.41785 (12)	0.0486 (6)	
H4A	0.1041	0.5699	0.4303	0.058*	
H4B	0.3742	0.5924	0.4580	0.058*	
C5	0.3526 (5)	0.7314 (4)	0.36991 (13)	0.0514 (6)	
H5A	0.3915	0.8629	0.3924	0.062*	
H5B	0.2255	0.7154	0.3325	0.062*	
C6	0.5811 (4)	0.7245 (3)	0.34417 (11)	0.0408 (5)	
H6	0.7122	0.7516	0.3820	0.049*	
C7	0.1191 (4)	0.1984 (3)	0.43585 (11)	0.0425 (5)	
C8	0.6621 (4)	0.8906 (3)	0.29618 (12)	0.0436 (5)	
H8A	0.5399	0.8620	0.2568	0.052*	
H8B	0.6794	1.0195	0.3171	0.052*	
C9	0.7391 (4)	0.7608 (4)	0.15427 (12)	0.0478 (6)	
C10	0.7786 (5)	0.5806 (4)	0.15077 (14)	0.0583 (7)	
H10	0.9218	0.5712	0.1731	0.070*	
C11	0.6055 (6)	0.4147 (5)	0.11411 (15)	0.0655 (8)	
H11	0.6334	0.2940	0.1116	0.079*	
C12	0.3903 (6)	0.4259 (5)	0.08091 (14)	0.0629 (7)	
C13	0.3540 (5)	0.6067 (5)	0.08475 (15)	0.0657 (8)	
H13	0.2106	0.6158	0.0624	0.079*	
C14	0.5257 (5)	0.7752 (4)	0.12102 (14)	0.0581 (7)	
H14	0.4985	0.8963	0.1231	0.070*	
C15	0.1990 (7)	0.2410 (6)	0.04226 (17)	0.0855 (11)	
H15A	0.2558	0.1300	0.0451	0.103*	0.50
H15B	0.1716	0.2706	−0.0036	0.103*	0.50
H15C	0.0481	0.2051	0.0608	0.103*	0.50
H15D	0.0612	0.2738	0.0231	0.103*	0.50
H15E	0.1454	0.1332	0.0717	0.103*	0.50
H15F	0.2689	0.1987	0.0074	0.103*	0.50
H4O	−0.145 (6)	0.081 (5)	0.4795 (18)	0.085 (11)*	

### Atomic displacement parameters (Å^2^)

**Table:**

	*U*^11^	*U*^22^	*U*^33^	*U*^12^	*U*^13^	*U*^23^
S1	0.0436 (4)	0.0492 (4)	0.0561 (4)	0.0181 (3)	0.0138 (3)	0.0168 (3)
O1	0.0410 (8)	0.0506 (9)	0.0490 (9)	0.0158 (7)	0.0070 (7)	0.0125 (7)
O2	0.0630 (12)	0.0484 (10)	0.0761 (12)	0.0226 (9)	0.0186 (10)	0.0222 (9)
O3	0.0395 (9)	0.0834 (14)	0.0793 (13)	0.0239 (9)	0.0234 (9)	0.0198 (10)
O4	0.0575 (11)	0.0653 (12)	0.0795 (13)	0.0268 (10)	0.0295 (10)	0.0342 (10)
O5	0.0615 (11)	0.0614 (11)	0.0751 (12)	0.0295 (9)	0.0257 (10)	0.0298 (9)
C1	0.0605 (15)	0.0417 (12)	0.0521 (13)	0.0198 (11)	0.0224 (11)	0.0060 (10)
C2	0.0639 (15)	0.0373 (12)	0.0632 (15)	0.0235 (11)	0.0254 (12)	0.0101 (10)
C3	0.0470 (12)	0.0366 (11)	0.0454 (12)	0.0136 (9)	0.0107 (10)	0.0065 (9)
C4	0.0595 (14)	0.0382 (12)	0.0528 (13)	0.0191 (11)	0.0206 (11)	0.0057 (10)
C5	0.0641 (15)	0.0374 (12)	0.0616 (14)	0.0237 (11)	0.0246 (12)	0.0111 (10)
C6	0.0454 (12)	0.0363 (11)	0.0419 (11)	0.0149 (9)	0.0104 (9)	0.0093 (8)
C7	0.0440 (12)	0.0367 (11)	0.0474 (12)	0.0138 (9)	0.0117 (10)	0.0049 (9)
C8	0.0446 (12)	0.0401 (11)	0.0502 (12)	0.0178 (10)	0.0143 (10)	0.0119 (9)
C9	0.0486 (13)	0.0572 (14)	0.0464 (12)	0.0277 (11)	0.0131 (10)	0.0120 (10)
C10	0.0613 (16)	0.0627 (16)	0.0602 (15)	0.0345 (13)	0.0065 (13)	0.0108 (12)
C11	0.085 (2)	0.0545 (16)	0.0618 (16)	0.0309 (15)	0.0104 (15)	0.0065 (12)
C12	0.0669 (18)	0.0685 (18)	0.0484 (14)	0.0174 (14)	0.0123 (13)	0.0065 (12)
C13	0.0554 (16)	0.085 (2)	0.0592 (16)	0.0309 (15)	−0.0002 (13)	0.0036 (14)
C14	0.0601 (16)	0.0658 (17)	0.0585 (15)	0.0349 (13)	0.0078 (12)	0.0094 (12)
C15	0.087 (2)	0.082 (2)	0.0660 (19)	0.0063 (18)	0.0072 (17)	−0.0036 (16)

### Geometric parameters (Å, °)

**Table:**

S1—O3	1.4226 (18)	C5—H5B	0.9700
S1—O2	1.4250 (19)	C6—C8	1.513 (3)
S1—O1	1.5678 (17)	C6—H6	0.9800
S1—C9	1.755 (3)	C8—H8A	0.9700
O1—C8	1.465 (3)	C8—H8B	0.9700
O4—C7	1.313 (3)	C9—C10	1.382 (4)
O4—H4O	0.89 (4)	C9—C14	1.387 (4)
O5—C7	1.216 (3)	C10—C11	1.378 (4)
C1—C2	1.524 (3)	C10—H10	0.9300
C1—C6	1.525 (3)	C11—C12	1.387 (4)
C1—H1A	0.9700	C11—H11	0.9300
C1—H1B	0.9700	C12—C13	1.377 (4)
C2—C3	1.518 (3)	C12—C15	1.514 (4)
C2—H2A	0.9700	C13—C14	1.383 (4)
C2—H2B	0.9700	C13—H13	0.9300
C3—C7	1.505 (3)	C14—H14	0.9300
C3—C4	1.531 (3)	C15—H15A	0.9600
C3—H3	0.9800	C15—H15B	0.9600
C4—C5	1.520 (3)	C15—H15C	0.9600
C4—H4A	0.9700	C15—H15D	0.9600
C4—H4B	0.9700	C15—H15E	0.9600
C5—C6	1.525 (3)	C15—H15F	0.9600
C5—H5A	0.9700		
			
O3—S1—O2	119.63 (12)	O4—C7—C3	113.7 (2)
O3—S1—O1	104.45 (11)	O1—C8—C6	108.88 (18)
O2—S1—O1	109.39 (11)	O1—C8—H8A	109.9
O3—S1—C9	109.50 (12)	C6—C8—H8A	109.9
O2—S1—C9	109.39 (12)	O1—C8—H8B	109.9
O1—S1—C9	103.13 (10)	C6—C8—H8B	109.9
C8—O1—S1	117.51 (13)	H8A—C8—H8B	108.3
C7—O4—H4O	110 (2)	C10—C9—C14	120.2 (3)
C2—C1—C6	111.4 (2)	C10—C9—S1	119.55 (19)
C2—C1—H1A	109.4	C14—C9—S1	120.1 (2)
C6—C1—H1A	109.4	C11—C10—C9	119.9 (2)
C2—C1—H1B	109.4	C11—C10—H10	120.1
C6—C1—H1B	109.4	C9—C10—H10	120.1
H1A—C1—H1B	108.0	C10—C11—C12	120.7 (3)
C3—C2—C1	111.5 (2)	C10—C11—H11	119.6
C3—C2—H2A	109.3	C12—C11—H11	119.6
C1—C2—H2A	109.3	C13—C12—C11	118.7 (3)
C3—C2—H2B	109.3	C13—C12—C15	121.2 (3)
C1—C2—H2B	109.3	C11—C12—C15	120.1 (3)
H2A—C2—H2B	108.0	C12—C13—C14	121.6 (3)
C7—C3—C2	112.18 (19)	C12—C13—H13	119.2
C7—C3—C4	109.70 (19)	C14—C13—H13	119.2
C2—C3—C4	111.24 (19)	C13—C14—C9	118.9 (3)
C7—C3—H3	107.8	C13—C14—H14	120.5
C2—C3—H3	107.8	C9—C14—H14	120.5
C4—C3—H3	107.8	C12—C15—H15A	109.5
C5—C4—C3	111.32 (19)	C12—C15—H15B	109.5
C5—C4—H4A	109.4	H15A—C15—H15B	109.5
C3—C4—H4A	109.4	C12—C15—H15C	109.5
C5—C4—H4B	109.4	H15A—C15—H15C	109.5
C3—C4—H4B	109.4	H15B—C15—H15C	109.5
H4A—C4—H4B	108.0	C12—C15—H15D	109.5
C4—C5—C6	112.09 (19)	H15A—C15—H15D	141.1
C4—C5—H5A	109.2	H15B—C15—H15D	56.3
C6—C5—H5A	109.2	H15C—C15—H15D	56.3
C4—C5—H5B	109.2	C12—C15—H15E	109.5
C6—C5—H5B	109.2	H15A—C15—H15E	56.3
H5A—C5—H5B	107.9	H15B—C15—H15E	141.1
C8—C6—C5	108.55 (18)	H15C—C15—H15E	56.3
C8—C6—C1	112.53 (19)	H15D—C15—H15E	109.5
C5—C6—C1	110.45 (19)	C12—C15—H15F	109.5
C8—C6—H6	108.4	H15A—C15—H15F	56.3
C5—C6—H6	108.4	H15B—C15—H15F	56.3
C1—C6—H6	108.4	H15C—C15—H15F	141.1
O5—C7—O4	122.8 (2)	H15D—C15—H15F	109.5
O5—C7—C3	123.5 (2)	H15E—C15—H15F	109.5
			
O3—S1—O1—C8	177.44 (16)	C5—C6—C8—O1	173.23 (17)
O2—S1—O1—C8	48.24 (18)	C1—C6—C8—O1	−64.2 (3)
C9—S1—O1—C8	−68.10 (17)	O3—S1—C9—C10	37.0 (2)
C6—C1—C2—C3	56.1 (3)	O2—S1—C9—C10	169.9 (2)
C1—C2—C3—C7	−178.2 (2)	O1—S1—C9—C10	−73.7 (2)
C1—C2—C3—C4	−54.9 (3)	O3—S1—C9—C14	−147.2 (2)
C7—C3—C4—C5	178.8 (2)	O2—S1—C9—C14	−14.3 (3)
C2—C3—C4—C5	54.1 (3)	O1—S1—C9—C14	102.0 (2)
C3—C4—C5—C6	−54.7 (3)	C14—C9—C10—C11	0.0 (4)
C4—C5—C6—C8	179.1 (2)	S1—C9—C10—C11	175.7 (2)
C4—C5—C6—C1	55.3 (3)	C9—C10—C11—C12	−0.5 (4)
C2—C1—C6—C8	−177.1 (2)	C10—C11—C12—C13	0.7 (5)
C2—C1—C6—C5	−55.6 (3)	C10—C11—C12—C15	−178.4 (3)
C2—C3—C7—O5	13.6 (3)	C11—C12—C13—C14	−0.4 (5)
C4—C3—C7—O5	−110.5 (3)	C15—C12—C13—C14	178.7 (3)
C2—C3—C7—O4	−167.2 (2)	C12—C13—C14—C9	−0.1 (4)
C4—C3—C7—O4	68.6 (3)	C10—C9—C14—C13	0.2 (4)
S1—O1—C8—C6	147.88 (16)	S1—C9—C14—C13	−175.4 (2)

### Hydrogen-bond geometry (Å, °)

**Table:**

*D*—H···*A*	*D*—H	H···*A*	*D*···*A*	*D*—H···*A*
O4—H4O···O5^i^	0.89 (4)	1.76 (4)	2.654 (3)	178 (3)

Symmetry codes: (i) −*x*, −*y*, −*z*+1.
